# Evolution of left ventricular strain after a first time myocardial infarction. A study using velocity encoded magnetic resonance imaging

**DOI:** 10.1186/1532-429X-11-S1-P268

**Published:** 2009-01-28

**Authors:** Ulrika Pahlm-Webb, Einar Heiberg, Erik Hedström, Hakan Arheden

**Affiliations:** Department of Clincial Physiology, Lund, Sweden

**Keywords:** Magnetic Resonance Image, Myocardial Infarction, Ischemia, Acute Myocardial Infarction, Regional Function

## Purpose

To compare the evolution of regional myocardial strain in an area of myocardial infarction to remote myocardium in patients with first time myocardial infarction.

## Introduction

After a myocardial infarction there is loss of function in the left ventricular region affected by ischemia. With time and treatment the function could improve. How fast the regional function improves is debated. Therefore we studied the evolution of left ventricular strain measured with velocity encoded MRI in the injured myocardium and compared it to strain in remote myocardium.

## Methods

Velocity-encoded magnetic resonance images (VE-MRI) were acquired in 8 patients (median age: 69 years, range: 41–74) with acute myocardial infarction and were followed with subsequent VE-MRI after 1 and 6 weeks, 6 and 12 months. The myocardium was manually delineated in the first time-frame and strain was automatically calculated using in house developed software http://segment.heiberg.se. Delayed enhancement images were used to determine the site of the myocardial injury. The first MRI was used as an internal control for each patient to be able to track change of regional strain over time. Strain in the injured myocardium was compared to remote myocardium over 12 months. A student t-test was used to test for significant change.

## Results

**Figure 1:**
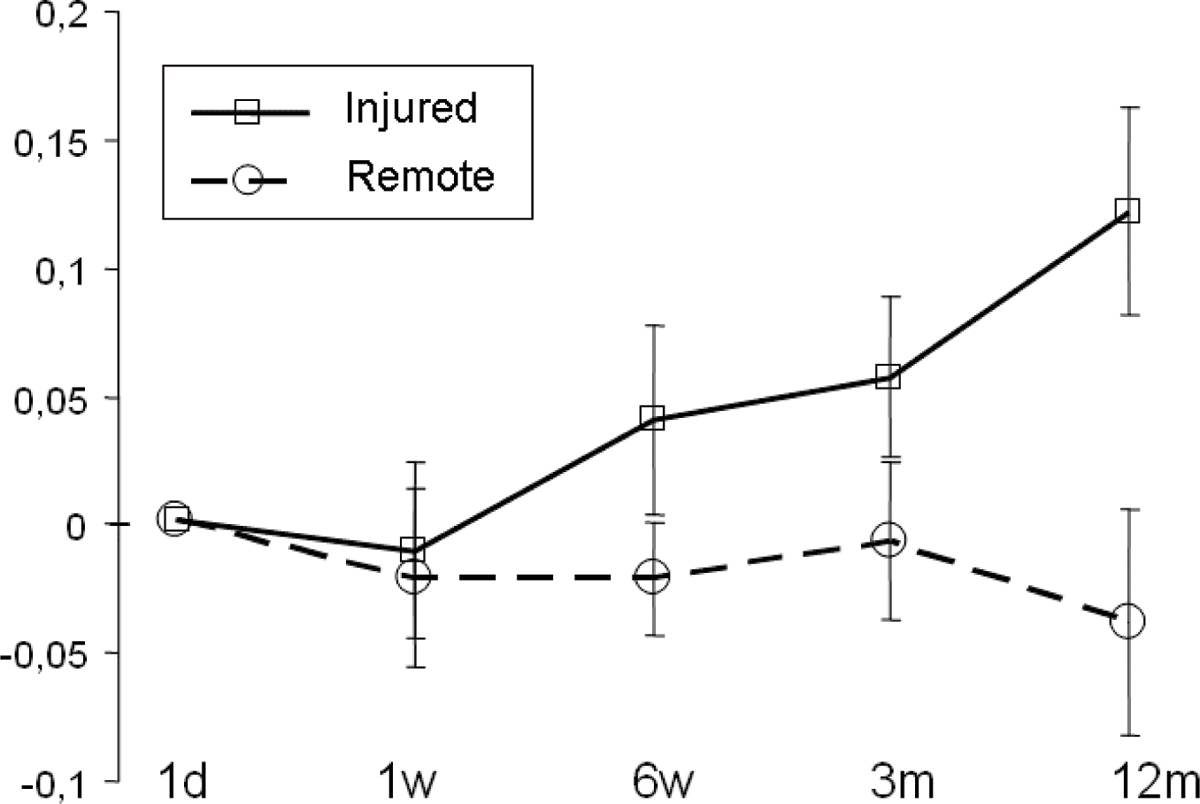
**Change of strain over time at one day, one week, six weeks, three months and one year, for injured and remote myocardium, respectively**.

Figure 1 shows change of strain over time at one day, one week, six weeks, three months and one year, for injured and remote myocardium, respectively. Regional strain in the injured region increases over 12 month (p < 0.05) while strain in remote myocardium decreases (p < 0.05).

## Conclusion

Regional strain in the injured region increases over 12 month while strain in remote myocardium decreases. This study shows a statistical difference in the change of strain 12 months after the infarct.

